# Role of augmented reality in surgery: editorial

**DOI:** 10.1097/JS9.0000000000001219

**Published:** 2024-02-16

**Authors:** Hitesh Chopra, Kavita Munjal, Sonia Arora, Shabana Bibi, Partha Biswas

**Affiliations:** a Department of Biosciences, Saveetha School of Engineering, Saveetha Institute of Medical and Technical Sciences, Chennai, Tamil Nadu; b Amity Institute of Pharmacy, Amity University, Noida, Uttar Pradesh; c Chandigarh Group of Colleges Landran, Kharar, Punjab, India; d Department of Biosciences, Shifa Tameer-e-Millat University, Islamabad, Pakistan; e Laboratory of Pharmaceutical Biotechnology and Bioinformatics, Department of Genetic Engineering and Biotechnology, Jashore University of Science and Technology, Jashore, Bangladesh

One cutting-edge gadget that might be useful in surgery is Augmented Reality (AR)^1^. Ultrasound, mammography, CT (computed tomography), and MRI (magnetic resonance imaging) scans, as well as other forms of real-time patient essential data, are very useful during surgical procedures. Typically, this information is shown on a 2D flat single screen. It’s inconvenient for doctors and nurses to constantly stare at a device that’s usually out of their line of sight. With the use of AR, a surgeon can see all of this information there in the operating room^1^. With an AR headgear, a surgeon would have instantaneous access to any and all digital files, photos, and data necessary for his work. Surgeons may see all pertinent data without taking their gaze off the patient.

While imaging has come a long way since 1950, the methods used to show pictures are mostly the same. Visual data is always shown on a 2D flat screen, on displays that take doctors’ and surgeons’ eyes off of the patient and off of their own hands. In addition, the pictures are shown from the point of view of the imaging equipment, rather than the viewer; hence, clinicians need to use ability and imagination to interpret and mentally project the images into the patient prior to and during treatments. Last but not least, clinicians need to pay extra attention to mentally merging numerous imaging types, such as angiography and CT, into a cohesive view of the patient since they are shown independently. Acquiring this talent requires years of training. Because of the partial and independent visibility of different types of visual input, clinicians may pay more steady attention to the total of a fused picture model. With this in mind, it seems that AR might be the most effective visualization technique for tackling problems of this kind. In order to make the operation simpler, safer, and more accurate, data displays are being shifted from 2D screens to real objects^2^.

The potential for AR to show imaging data and other patient information simultaneously has the potential to save lives and reduce medical mistakes. This is particularly true for out-of-the-OR treatments. When four or eight medical professionals are focused on caring for one patient in the operating room, the patient may be safer there than anywhere else in the hospital. Preoperative imaging ensures that all surgeries are well-organized. The anesthesiologist’s job is to keep an eye on the patient’s vitals while also providing pain relief and potentially life-saving drugs. In preparation for surgery, nurses always have everything on hand. It’s possible for surgeons to focus solely on their work. However, operating rooms are often fully occupied with elective patients, and room time is quite expensive. Every hospital relies on money from elective surgeries, so there is a lot of pressure to keep the operating rooms busy. In this context, quick, impromptu surgeries are difficult to do. Therefore, many of these treatments are carried out in ICUs (Intensive Care Units) and EDs (Emergency Departments) rather than in the operating room. These ‘bedside procedures’ may be the most dangerous for patients, but they also provide an opportunity for AR to shine^3^.

Spine surgeons may profit immensely from AR’s portability and image projection into the actual world via the use of portable overhead displays and image projections. Pedicle screwing is made easier with the use of AR during the operation. A spine surgeon does not have to take his eyes off the patient when he is utilizing the AR during surgery. Replacing lumbar pedicle screws is another surgical procedure that may benefit from 3D visualization and augmented reality. Spine surgery might benefit from real-time surgical guidance to increase precision and efficiency^3,4^.

Accurate imaging is particularly important in neurosurgery because of the field’s reliance on diagnostic imaging. The outcome of the operation depends on the doctor’s utilization of MRI and CT in preoperative planning and throughout intraoperative surgery. Neurological simulation and training are two new business uses for AR technology. It might significantly advance the profession and improve patient outcomes if used. The field of neurosurgery has been at the forefront of the surgical use of augmented reality. With the use of AR, neurosurgeons may see many 3D datasets superimposed in perfect harmony over the surgical site. Because of recent developments in imaging, display technology, registration, and robotic actuation, AR reality and 3D visualization are expected to play a significant role in neurosurgery in the near future. The introduction of 3D imaging technology and AR may increase the confidence of surgeons when executing delicate and demanding neurosurgery operations. While the neurosurgeons dissect and examine the patient’s anatomy, the instrument will provide virtual navigation to aid them. In the future, neurosurgical education at medical schools may use mixed reality, which combines the revolutionary technologies of augmented and virtual reality^5^.

The systems may cost upwards of hundreds of thousands of dollars per unit. It’s not that various image modes need dedicated screens, but rather that the industry standard is to offer expensive, incompatible bundles of hardware. The EMR (electronic medical records) system itself may function as a screen. The information from the hemodynamic monitors and the ventilators is not combined on one screen. AR may give a shared display, eliminating the need for several monitors to present different aspects of a patient’s data in real time. The medical field is one where AR can do out-of-the-box things. However, combining displays also come at costs, such as information overload and lack of visibility of details. Affordable and accessible health care for more people is possible because of technological advancements. There are several areas that might benefit from this technology, including education, illness monitoring and prevention, diagnostics, medical equipment maintenance and training, treatment and therapy planning, patient monitoring, and improved lifestyle and care. One day, these technologies may be utilized to help victims of road accidents or soldiers wounded in war. Many lives may be saved if the first responders on the ground provide HCP (health care provider)-guided first-aid therapy within the first few crucial minutes. Many AR-based devices have been approved by the FDA and are listed in Table 1. These listed FDA-approved devices are currently in use clinically.

**Table 1:** AR-based devices approved by FDA.

Submission number	Device	Company
K213034	SpineAR SNAP	Surgical Theater, Inc.
K221375	CureSight-CS100	NovaSight Ltd.
K220104	Knee+	Pixee Medical
K220733	OptiVu ROSA MxR	Orthosoft, Inc. (d/b/a Zimmer CAS)
K213684	SurgiCase Viewer	Materialise NV
K220146	VisAR	Novarad Corporation
K213751	NextAR TKA Platform My Knee PPS	Medacta International S.A.
K211254	ARAI Surgical Navigation System	Surgalign Spine Technologies
DEN210014	EaseVRx	AppliedVR, Inc.
K210344	inVisionOS	PrecisionOS Technology Inc.
K210859	NextAR Spine Platform	Medacta International, SA
DEN210005	Luminopia One	Luminopia, Inc.
K202927	EYE-SYNC	SyncThink, Inc.
K210726	ImmersiveTouch	ImmersiveTouch, Inc.
K211188	xvision Spine system (XVS)	Augmedics Ltd
K203115	ARVIS Surgical Navigation System	Insight Medical Systems Inc.
K210072	HOLOSCOPE-i	Real View Imaging Ltd.
K210153	NextAR RSA Platform	Medacta International SA
K202750	Knee+	Pixee Medical
K200384	HipXpert 3D Display and Anchoring Application	Surgical Planning Associates, Inc.
K202152	NextAR TKA Platform	Medacta International SA
K192890	SentEP	SentiAR, Inc.
K201465	SuRgical Planner (SRP) BrainStorm	Surgical Theater, Inc.
K193559	NextAR TKA Platform	MedactaInernational SA
K191014	Elements Viewer	Brainlab AG
K190929	xvision Spine system (XVS)	Augmedics Ltd.
K192186	I-Portal Neuro Otologic Test Center, I-Portal Video Nystagmography System, I-Portal Portable Assessment System – Nysragmograph	Neurolign USA, LLC
K183489	D2P	3D Systems, Inc.
K190764	SurgicalAR	MEDIVIS, Inc.
K183296	REAL Immersive System	Penumbra, Inc.
K182643	IRIS 1.0 System	Intuitive Surgical
K172418	OpenSight	Novarad Corporation
K170793	SuRgical Planner (SRP)	Surgical Theater, LLC
K162748	MindMotionPRO	MindMaze SA
K160584	Surgical Navigation Advanced Platform (SNAP)	SURGICAL THEATER, LLC
K153004	Clear Guide SCENERGY	CLEAR GUIDE MEDICAL
K151955	YuGo System	BIOGAMING LTD.
K152915	EYE-SYNC	SyncThink, Inc.
K142107	ECHO TRUE 3D VIEWER	ECHO PIXEL INC.

Source: https://www.fda.gov/medical-devices/digital-health-center-excellence/augmented-reality-and-virtual-reality-medical-devices

With the use of head-mounted displays, physicians may retain their focus on the patient while still having access to vital information, such as ultrasound scans and other health monitoring metrics, shown on the screens of various monitoring equipment, but they also block the vision of the doctor. It has the potential to one day eliminate the need for the different devices’ displays in favor of a unified image of the patient provided by head-mounted headgear. More and more doors are opening as a result of technological progress and forthcoming technologies such as 5G, the Internet of Things (IoT), and nanosensors. The widespread use of remote diagnostic and surgical procedures is made possible by the proliferation of high-speed networks. Medical researchers, scientists, mathematicians, physicists, data professionals, scientists, and computer programmers all worked tirelessly to bring AR to the present world. It will supposedly change the face of medicine and surgery as a whole because of its precision. Thanks to AR and 3D visualization, surgeons may concentrate on their work with surgical teams without having to worry about merging data from various patients^6^.

## Ethical approval

No ethical approval is required for this study.

## Consent

Not applicable.

## Sources of funding

No funding was received.

## Author contribution

H.C.: study concept and writing the manuscript; K.M. and P.B.: review and editing; S.A.: data analysis and writing the manuscript; S.B.: data collection and writing the manuscript.

## Conflicts of interest disclosure

The authors declare that they have no known competing financial interests or personal relationships that could have appeared to influence the work reported in this paper.

## Research registration unique identifying number (UIN)

Not applicable.

## Guarantor

Partha Biswas.
